# Post-mortem perinatal imaging: what is the evidence?

**DOI:** 10.1259/bjr.20211078

**Published:** 2022-04-25

**Authors:** Susan C Shelmerdine, Owen J Arthurs

**Affiliations:** 1 Department of Radiology, Great Ormond Street Hospital for Children NHS Foundation Trust, London, UK; 2 UCL Great Ormond Street Institute of Child Health, London, UK; 3 NIHR Great Ormond Street Hospital Biomedical Research Centre, 30 Guilford Street, Bloomsbury, London, UK; 4 Department of Radiology, St. George’s Hospital, Blackshaw Road, London, UK

## Abstract

Post-mortem imaging for the investigation of perinatal deaths is an acceptable tool amongst parents and religious groups, enabling a less invasive autopsy examination. Nevertheless, availability is scarce nationwide, and there is some debate amongst radiologists regarding the best practice and optimal protocols for performing such studies. Much of the published literature to date focusses on single centre experiences or interesting case reports. Diagnostic accuracy studies are available for a variety of individual imaging modalities (*e.g.* post-mortem CT, MRI, ultrasound and micro-CT), however, assimilating this information is important when attempting to start a local service.

In this article, we present a comprehensive review summarising the latest research, recently published international guidelines, and describe which imaging modalities are best suited for specific indications. When the antenatal clinical findings are not supported by the post-mortem imaging, we also suggest how and when an invasive autopsy may be considered. In general, a collaborative working relationship within a multidisciplinary team (consisting of radiologists, radiographers, the local pathology department, mortuary staff, foetal medicine specialists, obstetricians and bereavement midwives) is vital for a successful service.

## Introduction

Despite most parents being offered an autopsy after the loss of their child, only 30–40% consent to this examination,^1^ even though it is known to provide additional useful information in approximately half of all cases.^2–4^ Reasons for this refusal have included wanting to ‘protect’ their child from further “pain and harm”, but also religious and cultural beliefs.^5^ Without the information from an autopsy, many parents potentially miss out on important information which could help explain their pregnancy loss and potentially prevent future losses (*e.g.* if an inheritable diagnosis is uncovered). Some parents even regret their decision to forgo an autopsy many years later, feeling that many of their questions still remain unanswered.^6^ Unfortunately, until recently, alternative options for an invasive autopsy examination were unavailable to parents. Their choices were to either consent to the autopsy examination or to have no further investigations at all.

Some specialist centres have now supplemented this traditional ‘all or nothing’ approach with a range post-mortem imaging examinations providing what is known as a ‘less invasive autopsy’ (LIA) (or a ‘minimally invasive autopsy’ (MIA) where image-guided or laparoscopic surgical guided biopsies of organs are performed). The evidence for performing these examinations have focussed primarily on diagnostic accuracy trials, and comparisons between imaging modalities. Some specialist societies have even provided consensus expert opinions on how to perform various imaging tests for perinatal deaths and a few suggested guidelines have been proposed. For radiologists and clinicians interested in providing a post-mortem imaging/less invasive autopsy service, it can feel overwhelming in knowing where to start and what to do.

This article aims to condense the latest evidence for imaging perinatal deaths (*i.e.* foetal losses and early neonatal deaths < 7 days old) into a single document for ease of reference and simplify when to use them and what their limitations are. We have structured this article in a way that answers the most commonly asked questions we face from radiologists and parents regarding our own post-mortem imaging service with the intention of making this topic more accessible to a general audience. Given that forensic reasons are rarely the cause of death in the perinatal population, this article will not cover details regarding forensic imaging.

### What is the aim of perinatal post-mortem imaging?

The aim of perinatal post-mortem imaging is to provide additional information regarding the child’s demise, in a less invasive manner than conventional autopsy dissection. It is important to recognise that imaging does not replace all of the detailed analysis available following conventional autopsy, and a tissue biopsy or dissection may still be required to completely confirm or refute a diagnosis. However, imaging can clearly be used to help confirm or refute the clinical suspicions surrounding the cause of demise, and highlight discrepancies.

In developed countries, perinatal post-mortem imaging will predominantly involve clarifying antenatally suspected findings or highlighting missed internal anomalies that become strongly suspected after delivery (*e.g.* abnormal facies/discovery of polydactyly at external inspection, or disclosure of family history of consanguinity). As not all perinatal losses will be related to an abnormality with the foetus or neonate (*e.g.* maternal health issues, placental and cord abnormalities, obstetric complications), post-mortem imaging may not be required where an obvious alternative explanation exists.

Despite performing post-mortem imaging tests and potentially a full autopsy (including dissection), it is important to remember when counselling parents and clinicians that there remains a significant proportion of perinatal deaths in which the cause for foetal demise remains ‘undetermined’.^7,8^ Furthermore, some pathologies, such as pneumonia may mimic normal expected post-mortem changes (*e.g.* post-mortem-dependent changes in the lungs).^9^ These caveats are not necessarily reasons to refuse a request for post-mortem imaging (more to temper any unrealistic expectations), as some parents report feeling a sense of relief and reassurance even by an unremarkable result thereby absolving them of any blame for their loss.

### What is the best imaging modality to use for perinatal post-mortem imaging?

Different imaging modalities have different advantages and disadvantages and should be tailored according to the clinical scenario and availability of local resources and expertise.

**Figure 1.**
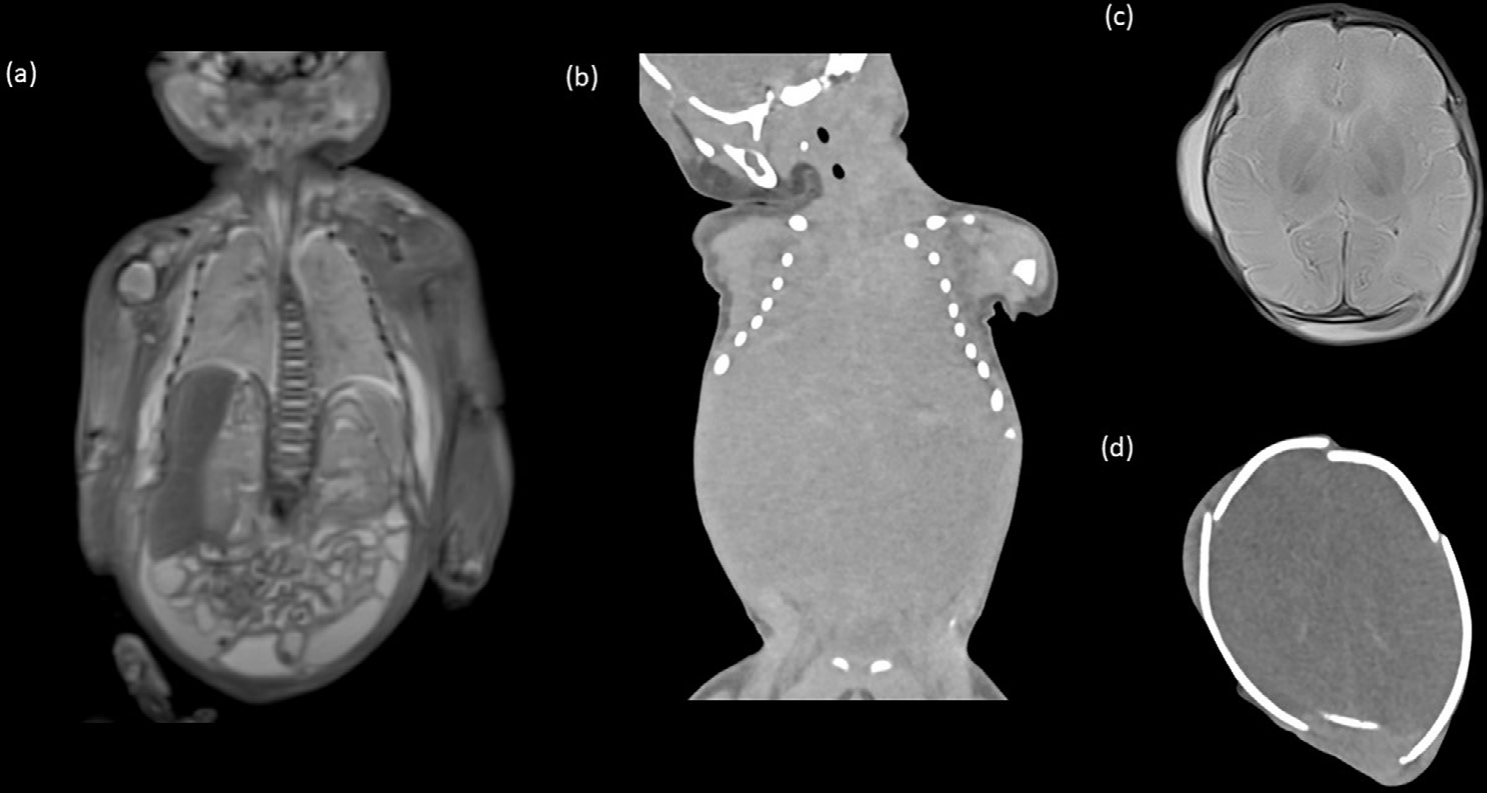
Comparison of post-mortem MRI and CT imaging in the same stillborn foetus of 36 weeks gestational age. Coronal post-mortem MRI (**a**) and CT (**b**) of the thorax and abdomen, with axial *T*_2_ weighted post-mortem MRI (**c**) and axial CT (**d**) of the brain are demonstrated. There were no congenital abnormalities seen on antenatal or post-mortem imaging, however, it is clear that the non-contrast MRI allows for better internal soft tissue differentiation compared with the CT imaging. For this reason, post-mortem CT is not recommended as a routine tool for non-forensic perinatal post-mortem imaging.

Where the perinatal loss is due to a natural cause (*i.e.* not a forensic referral), then the gestational age and patient size will commonly dictate the appropriate modality, largely due to issues related to image resolution. In general, for mid-second and third trimester perinatal losses (*i.e.* >20 weeks’ gestation), whole-body post-mortem ultrasound (PMUS) or MRI (PMMR) are the most appropriate tools. This is in contrast with adult post-mortem imaging where CT is the commonest modality. For perinatal deaths, the lack of internal soft tissue contrast makes CT less helpful (Figure 1), although it is an excellent tool for identifying bony injuries in suspected trauma.^10^

Smaller foetuses, weighing less than 500 g (post-mortem body weight) or aged less than 18 weeks’ gestation are more challenging to image with standard imaging technology.^11^ In these cases, high field MRI (>7 T) or ‘microfocus computed tomography’ (*i.e.* micro-CT)^12^ may be more applicable although these tools are not currently widely available. Where referral to a specialist centre is unavailable, then parents should be informed that although PMUS or PMMR can be offered, a higher rate of non-diagnostic information will be expected.

**Figure 2.**
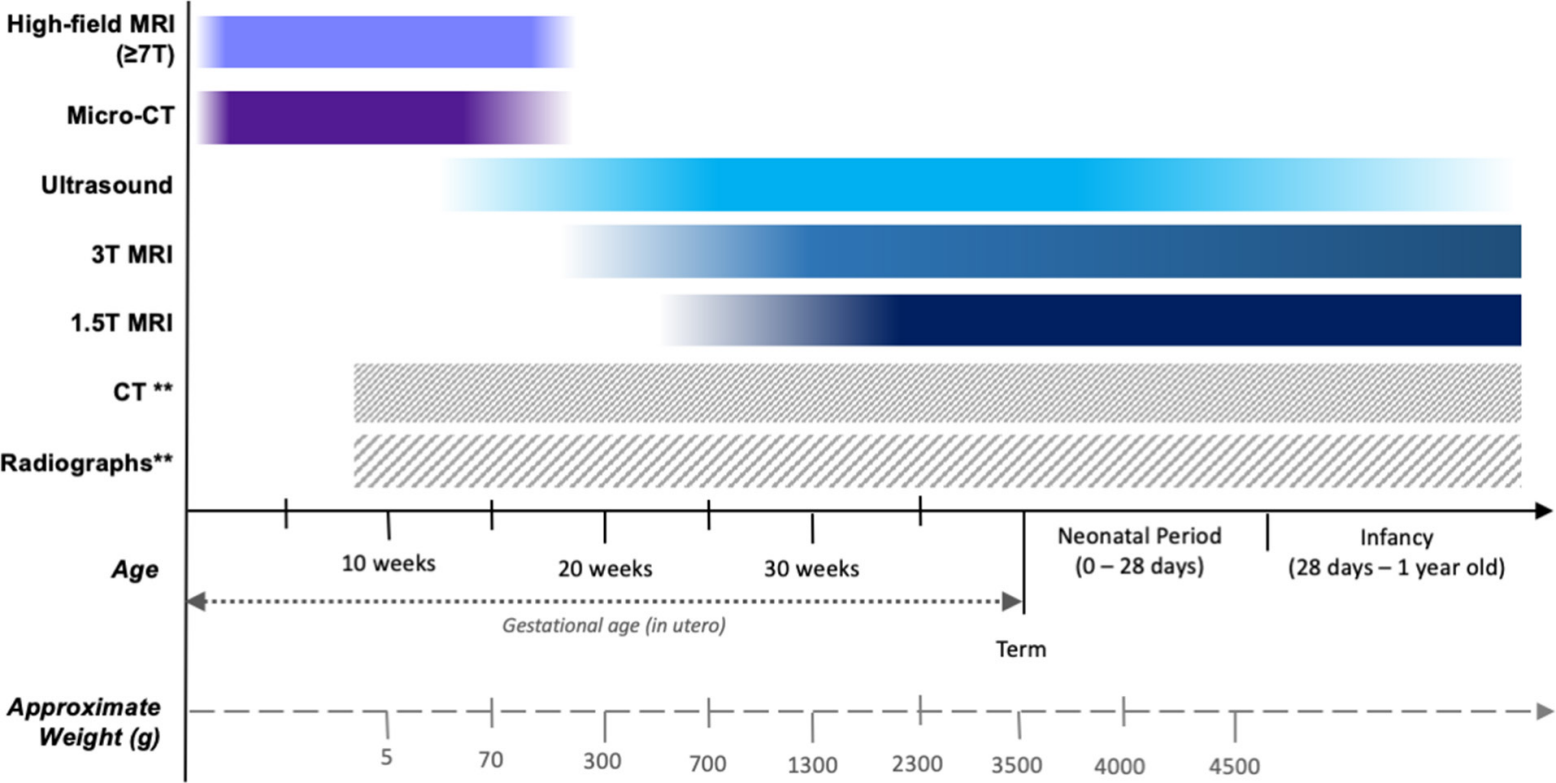
Typical estimated gestational ages and the approximate post-mortem weights (**g**) at which various post-mortem imaging modalities would provide diagnostic quality examinations. ** Technically, radiographs and CT can be performed at any age after 8 weeks gestation (when the foetal skeleton beings to ossify), but in practice they are best reserved for specific clinical situations, such as for suspected skeletal abnormalities or trauma.^14^ *Reproduced from Shelmerdine SC et al, Insights into Imaging 2021.* ^13^

A quick ‘at a glance’ reference tool is provided in Table 1 summarising the different advantages of post-mortem imaging modalities, and Figure 2 provides a visual guide showing which imaging modalities are most likely to be diagnostic at different gestational ages and sizes.

**Table 1.** Benefits and drawbacks of different post-mortem imaging modalities for perinatal loss

	Radiographs	Ultrasound	CT	MRI (3 T or 1.5 T)	Micro-CT	High Field MRI (7 T+)
**Availability**	Easily available	Easily available	Easily available	Moderate	Limited: few select centres/research facilities	Limited: few select centres/research facilities
**Cost**	Cheap	Cheap	Cheap	Expensive+	Same cost as CT scanner.	Expensive+ +
**Size of foetus**	Any size	Any size – although intrauterine retention time may affect image quality.	Technically feasible, but poor diagnostic accuracy and lack of internal contrast from lack of body fat.	Better for larger foetuses, poorer for body weight <500 g.	Up to 30 cm in length, limited by scanner bore.	Similar to micro-CT
**Advantages**	Easy to perform, already part of routine autopsy service.	Ease of access, cheap and portable. Facilitates image- guided biopsies.	Highest accuracy for intracranial and musculoskeletal trauma (older children; trauma)	Multiple sequences, multiplanar reconstructions	Excellent resolution & soft tissue detail. Excellent bone detail without exogenous contrast.	Excellent resolution & soft tissue detail No need for exogenous contrast
**Drawback**	No internal soft tissue detail. Only useful in minority (<5%) of cases	Operator-dependent Requires a hands-on approach (radiologist).	Poor soft tissue detail due to lack of internal body fat.	Availability/access may be limited Poorer resolution in smaller foetuses.	Iodine contrast is required for soft tissue detail, which can cause tissue discoloration.	Expensive, limited access, long scanning times (hours).
**Indication:**	Estimation of foetal gestational age, diagnosis of skeletal dysplasias and limb anomalies	Assessment of soft tissue/internal organ detail	Bony injuries; trauma; consider for skeletal dysplasias or trauma (although radiographs better and cheaper)	Assessment of soft tissue/internal organ detail	Small foetuses (<20 weeks gestation) where ultrasound and 1.5 T/3 T MRI non-diagnostic.	Currently research tool only.

Reproduced from Shelmerdine SC et al, Insights into Imaging 2021.^13^

### When should the post-mortem imaging happen?

The time interval between foetal delivery (or perinatal death) and post-mortem imaging (the so-called ‘post-mortem interval’) has not been widely shown to contribute to the post-mortem imaging quality. Nevertheless, some researchers advocate performing the imaging as soon as reasonably possible (ideally within 3 days of the death/demise) to avoid further organ autolysis and to delays in providing a diagnosis for the bereaved families. Imaging within 24 h may be desirable but will not be achievable for all centres who depend on retrieval and storage of the body elsewhere.

Several studies have shown the degree of maceration to be more important in determining image quality, *i.e*. time between an intrauterine foetal death and delivery—also known as the ‘intrauterine retention time’. This has been reported to be the most significant factor in acquiring a diagnostic quality post-mortem ultrasound study^15,16^ as the degree of tissue breakdown increases with a longer intrauterine retention time making it difficult to differentiate soft tissue planes. One study found that post-mortem ultrasound brain imaging was non-diagnostic in less than 10% when completed within 12 h of the foeticide (and delivery), but greater than 40% non-diagnostic when performed at 48 h or more.^16^ In these situations, it may be preferential to perform MRI in extensive maceration, although this may also yield suboptimal imaging if the intrauterine retention time has been >24 h and a brain abnormality is suspected.^17^

### What is the diagnostic accuracy of the post-mortem imaging modalities?

From a review of the available evidence published on the PubMed, Embase and Google Scholar databases (using the search terms ‘post-mortem’, ‘autopsy’ with ‘imaging’ and ‘perinatal’, ‘foetal’ or ‘neonatal’) the selected studies below for each modality have been chosen as being representative of the wider literature, based on articles with the largest sample sizes and a preference towards systematic reviews or studies comparing two or more modalities allowing for comment on differences in diagnostic accuracies to be made.

#### MRI

Historically, the largest prospective paediatric post-mortem imaging study to date (the ‘MARIAS’ study,^18^ including 277 perinatal losses found >90% concordance for overall diagnosis compared to standard autopsy (sensitivity 89.7%, specificity 95%). This was particularly high for abnormalities of the heart, brain and musculoskeletal system. Whilst this was a single centre study, similarly high diagnostic accuracy rates have been found on studies assessing other perinatal populations.^19–21^ Furthermore, where available, performing post-mortem MRI at higher field strengths (*e.g.* 3 T) has resulted in higher concordance rates for overall diagnosis with autopsy than standard 1.5 T MRI^22^ (77% *vs* 69% respectively). MRI can give clinically useful information where neuropathology is non-diagnostic^23^ due to tissue autolysis. Nevertheless, recent publications now suggest that antenatal (foetal) brain MRI is better for diagnosing complex neurological conditions than post-mortem MRI.^24^

#### Ultrasound

Factors for non-diagnostic ultrasound imaging have been described above. When the imaging is of diagnostic quality then diagnostic accuracy rates similar to both 1.5 T^25^ and 3 T MRI^26^ have been reported, with an estimated overall sensitivity of 73% and specificity 97% (based on a systematic review of 455 perinatal losses).^27^ The highest sensitivity rates were found for brain imaging (84%), and lowest for cardiothoracic abnormalities (51%).

#### CT

In a subset of cases that underwent both 1.5 T MRI and CT (*n* = 82),^10^ CT generated more non-diagnostic studies (22% *vs*  5%) and the overall accuracy rate was also lower (59% *vs*  63% where both CT and MRI studies were of diagnostic quality). For these reasons, CT is rarely performed in perinatal post-mortem imaging, but may be more useful in children, particularly following suspected trauma.^28,29^

#### Micro-CT

Two of the largest case series published comparing foetal micro-CT with standard autopsy^12,30^ demonstrated high sensitivity and specificity rates for overall diagnosis (94–100% sensitivity, 90–100% specificity).^12,30^ However, this technique requires pre-imaging tissue preparation with an iodinated contrast medium to improve soft tissue differentiation. This can result in residual discolouration of the foetus and potential tissue shrinkage,^31^ although neither of these have caused significant concerns in current clinical use.^32^ Furthermore, the pre-imaging ‘staining’ process may take several days introducing further steps to the imaging process and potential delays in ‘turnaround’ times, with limited availability and expertise currently.^32^

#### High-field MRI

A recent systematic review^33^ only found three publications where high-field MRI was used for whole body post-mortem foetal imaging (7–11 T). The largest of these studies^34^ (*n* = 17), found a complete agreement between 9.4 T MRI and standard autopsy for overall diagnosis. Contrast staining of the foetus is not required for any MRI imaging (unlike for micro-CT),^35^ but long scan times are often required.

#### When would additional tissue sampling (or autopsy) be required?

In 60% of intrauterine deaths, despite a full ‘invasive’ autopsy, the foetal death is unexplained.^7^ Where a cause is found, this is frequently through non-invasive means; *e.g.* 38% intrauterine deaths are already explained from placental and clinical assessment before any invasive dissection.^7^ Outcomes from >5000 paediatric autopsies recently showed that acquiring histological tissue would only provide the cause of death in a minority of perinatal cases (<1%) where the death is not explained via non-invasive means (*i.e.* undetermined from placental, clinical or imaging examinations).^36^ In other words, where the post-mortem imaging does not demonstrate any underlying macroscopic abnormality, there is a low likelihood that microscopic tissue sampling is of any benefit. In addition, it has been reported that where antenatal ultrasound and post-mortem MRI results agree (either on absence or presence of an abnormality), then the additional value of an autopsy is low (<5%).^37^ Therefore, it could be argued that post-mortem imaging could be used to triage cases, and invasive autopsy should be reserved only for those cases in whom post mortem imaging demonstrates an unsuspected abnormality, is non-diagnostic, or reveals a discordance between antenatal and post-mortem imaging. This change in service delivery would generate the highest yield from both post-mortem imaging and autopsy alike, and could be effective where limited resources are available.

Should tissue sampling be required, minimally invasive methods are now available using image guidance. This is preferable over ‘blind’ percutaneous needle biopsies that use surface landmarks to locate organs given the low success rates in acquiring the intended tissue (<52%). Ultrasound-guided biopsies are more successful for this task (76.1%), and can be performed via the umbilical vein avoiding any incisions to the body (so-called the ‘INTACT’ biopsy procedure).^38^ Laparoscopically guided tissue sampling yields the highest success rates (>80%)^39^ but can be difficult to perform in small foetuses, and require small incisions to the body and expensive equipment not found in many mortuaries. It is vital prior to conducting any tissue sampling to remember that additional parental consent should have been provided for this. Where that is not the case, but additional tissue sampling may be beneficial, further counselling and discussions should take place before proceeding.

#### Are there published imaging protocols for post-mortem imaging?

Several recommended protocols have been published and endorsed by international societies. For PMMR, the European Society of Paediatric Radiology (ESPR) has endorsed a suitable MRI protocol which can be completed in 30–60 min, devised via an expert consensus survey.^40^ A more abbreviated protocol (lasting less than 20 min) has been suggested should MRI scanner time be particularly limited.^41^ Where readers wish to review a more comprehensive article on the different post-mortem MRI sequences, then the following reference is highly recommended.^42^

With regards to post-mortem CT imaging, the ESPR (together with the International Society for Forensic Radiology and Imaging, ISFRI) have published imaging recommendations,^43^ although this modality is more typically applied to forensic childhood cases rather than perinatal deaths. Where post-mortem ultrasound is being performed, how to conduct, report and recognise common developmental pathologies has been published in detail.^44,45^

For readers wishing to develop post-mortem micro-CT, a step-by-step guide has been published.^32^ There is no recommended protocol for high-field MRI although a few research teams have described their methodology which could be replicated if desired.^33,46^

#### Are there published guidelines for which imaging tool to use?

There are no internationally recognised referral guidelines for perinatal post-mortem imaging, although the Royal College of Pathologists (RCPath) do make reference to post-mortem imaging tools in their perinatal autopsy guidelines,^47,48^ stating that in addition to post-mortem radiographs (*i.e.* skeletal surveys), MRI and micro-CT imaging may be of value. Post-mortem ultrasound is not included in these guidelines. The British Neuropathological Society (BNS) and International Society of Forensic Imaging (ISFRI) have published a joint consensus statement regarding when post-mortem neurological imaging may be helpful.^49^ In this, they suggest post-mortem imaging of the brain where there is a high likelihood that the brain may be autolysed and damaged by autopsy extraction, and to help confirm diagnoses resulting in a termination of pregnancy. The modality of choice should be determined by the radiologist and pathologist involved in the case.

In the Netherlands, the Dutch national guidelines^50^ for foetal and neonatal death investigation recommend post-mortem imaging for stillbirths (with normal antenatal imaging), for terminations of pregnancy (where confirmation of abnormal findings is required and autopsy is refused) and where abnormalities were detected antenatally and foetal demise ensued. In these cases, post-mortem MRI is the modality of choice. Where parents already consent to an autopsy, or where the confirmation of abnormal antenatal imaging findings is not required, then post-mortem imaging is not performed.

Kang et al^51^ have published a flowchart asking referrers whether antenatal imaging demonstrated any structural abnormalities, and whether a genetic diagnosis has already been provided. Where there are no antenatally suspected anomalies or genetic cause for the foetal demise then they suggest performing post-mortem imaging. Where the antenatal imaging was abnormal (without a genetic cause) then an invasive autopsy was favoured over post-mortem imaging.

**Figure 3.**
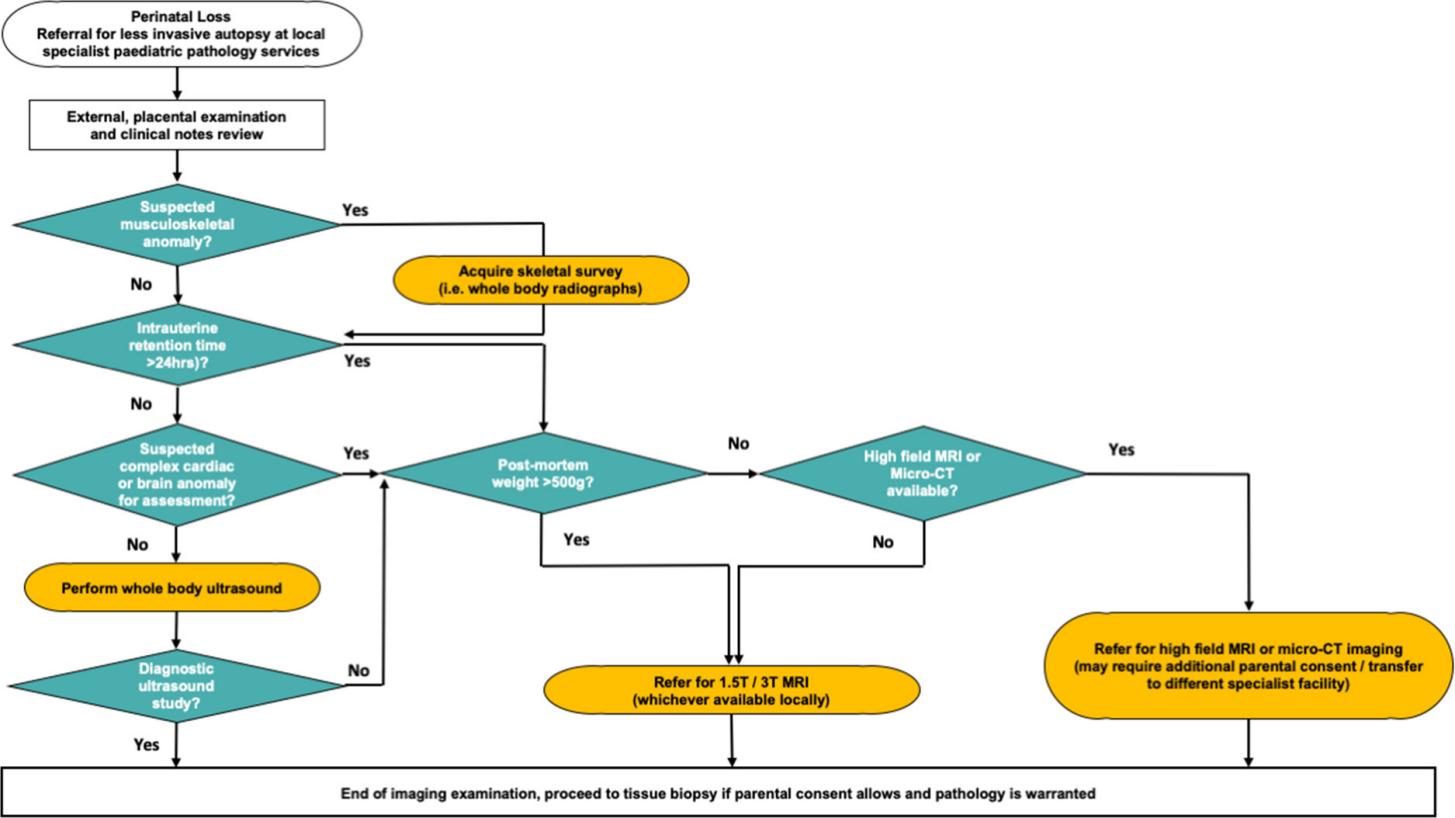
Recommended post-mortem imaging flowchart for non-invasive investigation of perinatal loss. Small foetuses present a challenge for post-mortem imaging, and care should be taken when interpreting imaging results in this cohort. A foetus weighing over 500 g provides the greatest likelihood for a diagnostic quality 1.5 T MRI study,^11^ and those weighing <300 g are best suited for micro-CT or high-field MRI.^33^ Where neither MRI nor micro-CT are available, ultrasound may be attempted but there is a higher likelihood of a false or non-diagnostic result.^26,52^ Foetuses weighing between 300 and 500 g have been reported to take >7 days to iodinate, and therefore delay micro-CT imaging. If available, 3 T MRI could be attempted for this foetal cohort.^22^ *Reproduced from Shelmerdine et al, Insights into Imaging 2021.* ^13^

Our suggested general approach (outlined in Figure 3), presumes that the decision for post-mortem imaging has already been made between the parents and referring clinician (based on parental refusal for autopsy and availability of an imaging service). We outline a decision-making process to help radiologists determine which type of post-mortem imaging is most ideal to perform (assuming that all resources and expertise are available to them),^13^ and also provide alternative options where more specialist equipment (*e.g.* micro-CT or high-field MRI scanners) is unavailable.

#### How to report and train in perinatal post-mortem imaging?

At present, there are no formalised paediatric post-mortem imaging fellowships available, although the ESPR do run an annual paediatric post-mortem imaging workshop (available via their website www.espr.org), and both the ESPR and ISFRI have dedicated paediatric post-mortem imaging subcommittees which can provide advice regarding training opportunities and informal visits with specialist centres that regularly perform post-mortem imaging.

Several publications are recommended for radiologists wishing to familiarise themselves with normal and abnormal appearances at post-mortem imaging at post-mortem MRI,^9,53^ ultrasound^44,45^ and CT,^54^ and a reporting template has been provided in the supplementary materials section of this reference.^15^

## Conclusions

Perinatal post-mortem imaging provides a less invasive means by which to provide information regarding causes for foetal and neonatal deaths. Post-mortem imaging tools (*e.g.* ultrasound) may also assist in guiding tissue biopsies or a more limited invasive autopsy (possibly of just one body organ or system) where needed. The main advantages and drawbacks of a variety of modalities have been addressed in this article, with the current understanding of their diagnostic accuracy, which have influenced the suggested flowchart. Key aspects from the perinatal history crucial to these decisions include gestational age, known or suspected antenatal anomalies, as well as logistical details regarding the availability of different scanners locally.
